# Supplementary material for: Novel COX11 Mutations Associated with Mitochondrial Disorder: Functional Characterization in Patient Fibroblasts and Saccharomyces cerevisiae
Source: Int J Mol Sci. 2023 Nov 23;24(23):16636. doi: 10.3390/ijms242316636 (PMC10706101; doi:10.3390/ijms242316636)
Supplement: Supplementary file 1 [file ijms-24-16636-s001.zip › Suppl Figures_final.pptx]

## Slide 1
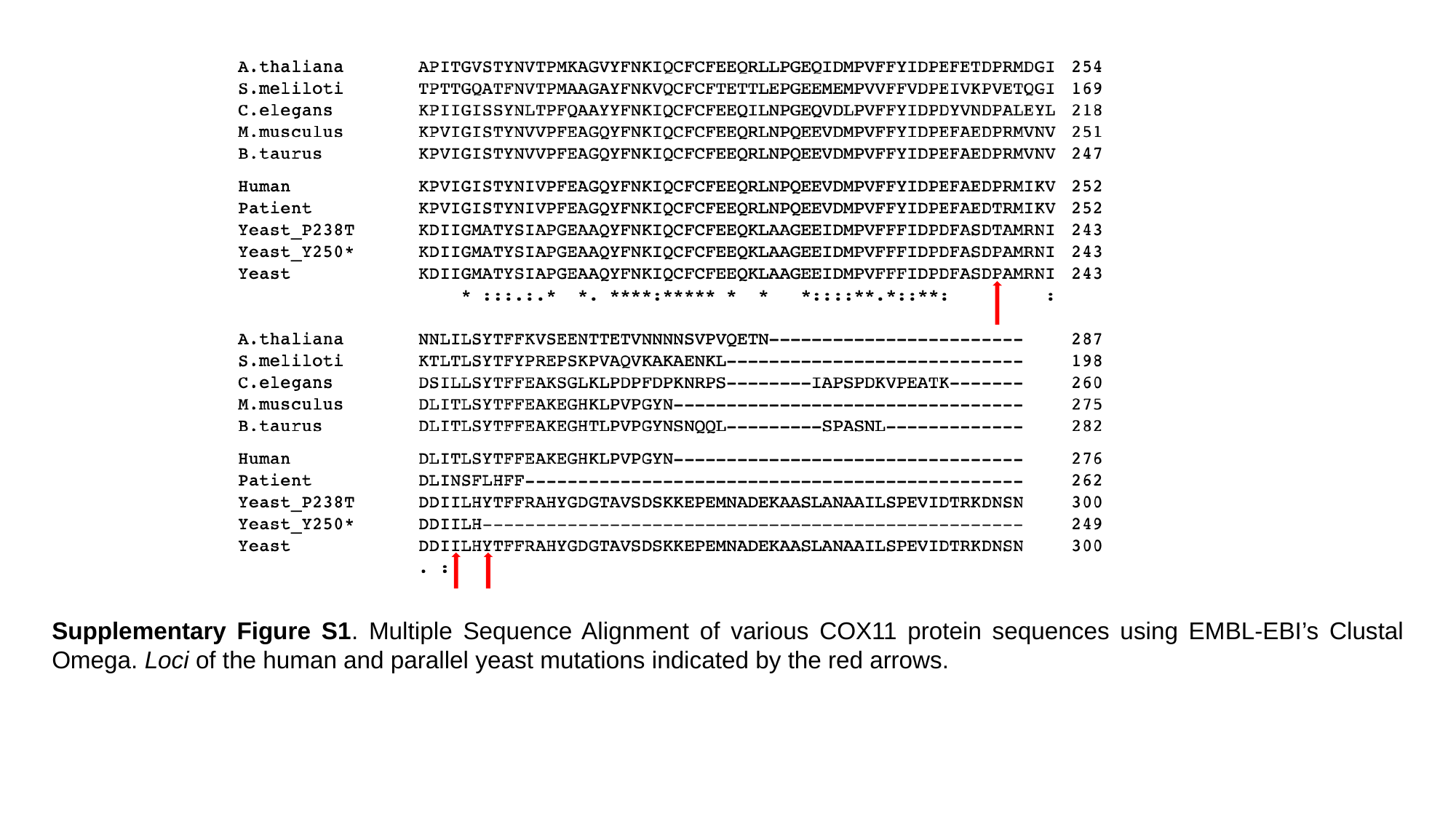

Supplementary Figure S1. Multiple Sequence Alignment of various COX11 protein sequences using EMBL-EBI’s Clustal Omega. Loci of the human and parallel yeast mutations indicated by the red arrows.

## Slide 2
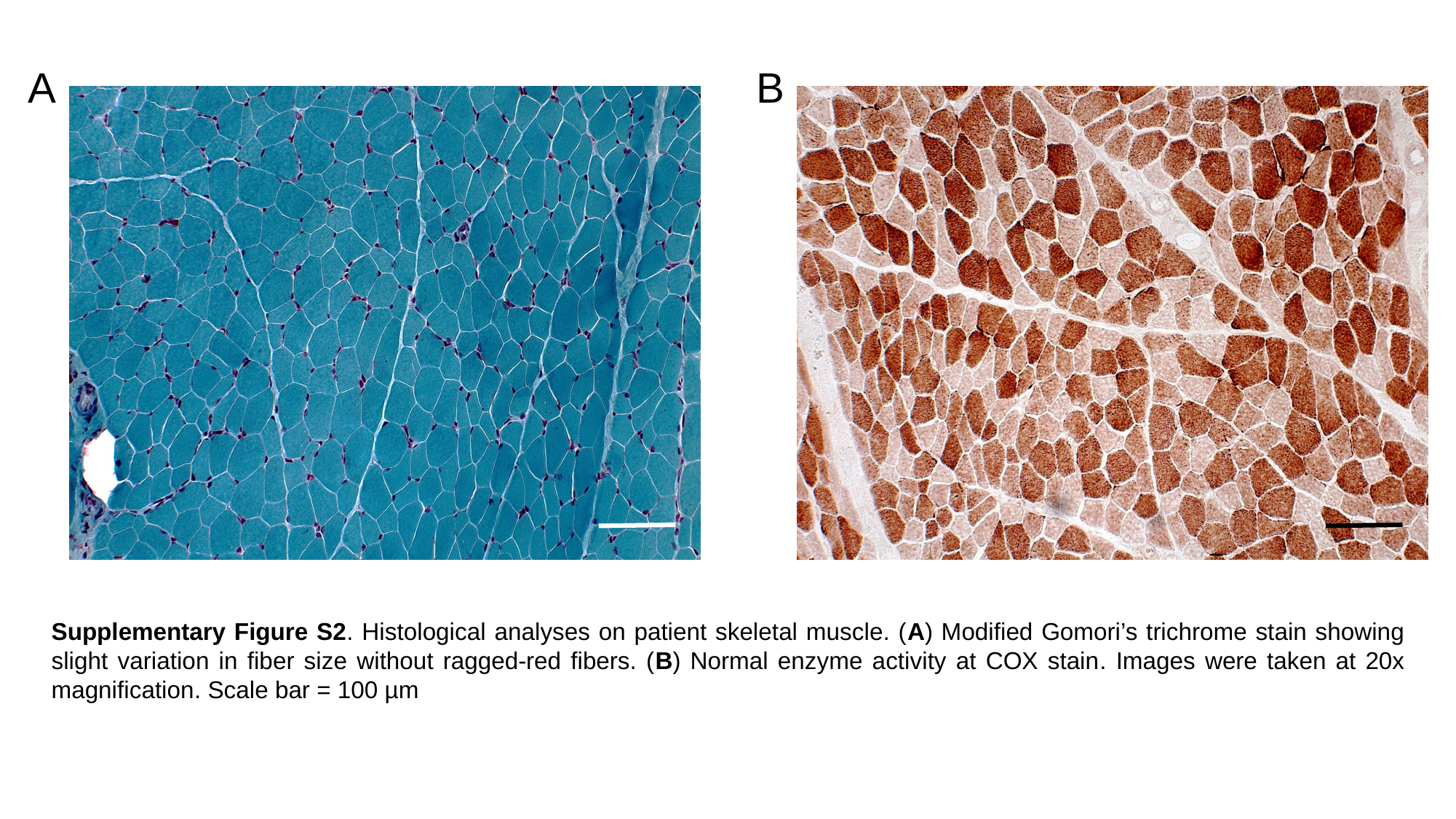

A
B
Supplementary Figure S2. Histological analyses on patient skeletal muscle. (A) Modified Gomori’s trichrome stain showing slight variation in fiber size without ragged-red fibers. (B) Normal enzyme activity at COX stain. Images were taken at 20x magnification. Scale bar = 100 µm

## Slide 3
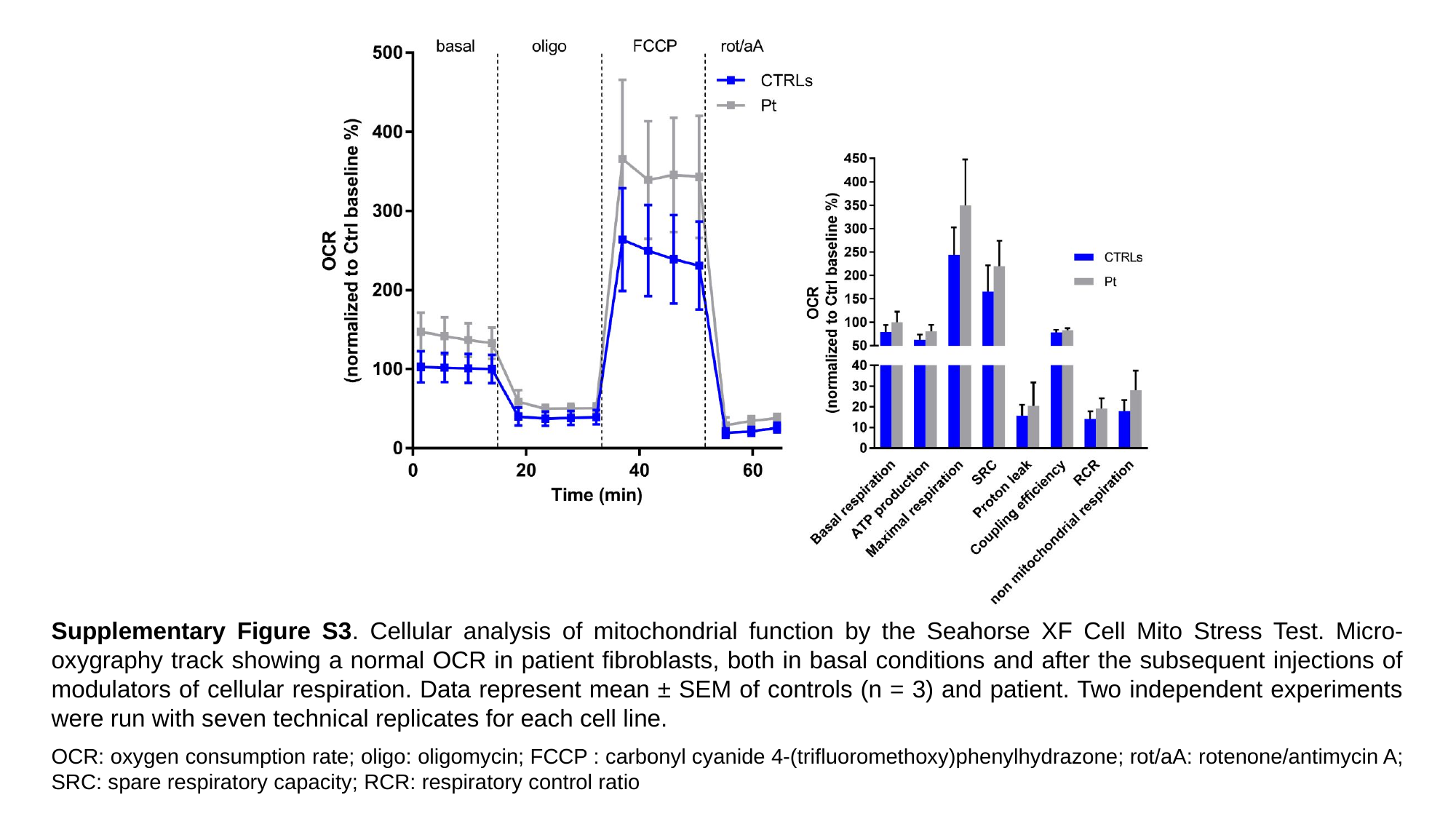

Supplementary Figure S3. Cellular analysis of mitochondrial function by the Seahorse XF Cell Mito Stress Test. Micro-oxygraphy track showing a normal OCR in patient fibroblasts, both in basal conditions and after the subsequent injections of modulators of cellular respiration. Data represent mean ± SEM of controls (n = 3) and patient. Two independent experiments were run with seven technical replicates for each cell line.
OCR: oxygen consumption rate; oligo: oligomycin; FCCP : carbonyl cyanide 4-(trifluoromethoxy)phenylhydrazone; rot/aA: rotenone/antimycin A; SRC: spare respiratory capacity; RCR: respiratory control ratio
