# Supplementary material for: Novel COX11 Mutations Associated with Mitochondrial Disorder: Functional Characterization in Patient Fibroblasts and Saccharomyces cerevisiae
Source: Int J Mol Sci. 2023 Nov 23;24(23):16636. doi: 10.3390/ijms242316636 (PMC10706101; doi:10.3390/ijms242316636)
Supplement: Supplementary file 1 [file ijms-24-16636-s001.zip › Supplementary Tables.pdf]

Supplementary Table S1

Plasma amino acids and urine organic acids alterations in our case at the age of 23 months.

	**This case**	**Normal value**
**Plasma amino acids**		
Alanine	520 umol/l	167-389
Valine	381.33 umol/l	140-302
Isoleucine	100.26 umol/l	31-88
**Urine organic acids**		
Lactate	235 umol/mmol	< 50
Pyruvate	305 µmol/mmol	< 20
Ethylmalonic acid	39 µmol/mmol	< 20
Succinic acid	98 µmol/mmol	< 20
Fumaric acid	118 µmol/mmol	< 10
Glutaric acid	202 µmol/mmol	< 10
2-OH-glutaric acid	165 µmol/mmol	< 20
Ketoglutaric acid	1200 µmol/mmol	< 120

Supplementary Table S2

In silico pathogenicity predictions for *COX11* mutation c.739C>A (p.P247T)

***In silico* tool**	**Predictions**
CADD	deleterious (26.1)
MetaRNN	damaging (0.8927)
REVEL	pathogenic (0.5889)
PolyPhen-2	probably damaging (0.999)
SIFT	damaging (0.003)
MutationTaster	disease causing (1)
EIGEN	pathogenic (0.8179)

CADD (http://cadd.gs.washington.edu/); MetaRNN (http://www.liulab.science/metarnn.html) REVEL (https://sites.google.com/site/revelgenomics/); PolyPhen-2 (http://genetics.bwh.harvard.edu/pph2); SIFT (https://sift.bii.a-star.edu.sg/index.html); Mutation Taster (http://www.mutationtaster.org); EIGEN (http://www.columbia.edu/~ii2135/eigen.html).

Supplementary Table S3

Spectrophotometric determination of RC activities in patient skeletal muscle

Enzyme	Activity	Normal Range
NADH:ubiquinone oxidoreductase (Complex I)	25.06 (2.01)	27.5-39.5 (1.56-2.60)
Succinate dehydrogenase (Complex II)	1.82 (0.15)	1.2-2.0 (0.05-0.105)
NADH cytochrome c oxidoreductase (Complexes I+III)	2.14 (0.17)	0.65-1.50 (0.11-0.25)
Succinate cytochrome c reductase (Complexes II+III)	0.56 (0.04)	0.45-0.90 (0.05-0.08)
Cytochrome c oxidase (Complex IV)	2.89 (0.23)	1.80-2.45 (0.17-0.28)
Citrate synthase (CS)	12.44	8.91-15.00

Activities are expressed in nmol/min/gr tissue. In brackets the values normalized to the activity of CS
